# On-site personal protective equipment signage and use by road construction workers in Ghana: a comparative study of foreign- and locally-owned companies

**DOI:** 10.1186/s12889-021-12376-2

**Published:** 2021-12-23

**Authors:** Isaac Kofi Yankson, Nana Kwame Nsiah-Achampong, Paul Okyere, Francis Afukaar, Easmon Otupiri, Peter Donkor, Charles Mock, Ellis Owusu-Dabo

**Affiliations:** 1 Council for Scientific and Industrial Research-Building and Road Research Institute (CSIR-BRRI), Kumasi, Ghana; 2 School of Public Health, Kwame Nkrumah University of Science and Technology, CSIR-BRRI, P.O. Box UP40, Kumasi, Ghana; 3 School of Medicine and Dentistry, Kwame Nkrumah University of Science and Technology, Kumasi, Ghana; 4 Department of Surgery, Komfo Anokye Teaching Hospital, Kumasi, Ghana; 5 University of Washington, Seattle, WA USA

**Keywords:** Occupational safety, road construction, personal protective equipment, foreign-owned companies, locally-owned companies, Ghana

## Abstract

**Background:**

Road construction work has specific risks and safety issues which have not been adequately addressed in most low- and middle-income countries, especially Africa. The objective of this study was to determine the prevalence of personal protective equipment (PPE) use during road construction activities by workers in foreign- owned against locally-owned road construction companies in Ghana.

**Methods:**

An institution-based cross-sectional survey was undertaken during January – March, 2020 to study 389 road construction workers who were actively working on site. They were unobtrusively observed to capture whether or not they wore the appropriate PPE at the time of the survey. The PPE of interest were: hard hat, goggles, shoes, nose masks, hearing protection, gloves and reflective vests/apparel. On-site posted PPE signage was also checked.

**Results:**

Majority of workers were males (96.9%) and labourers (53.5%). Similar numbers of workers in locally-owned (195) and foreign-owned (194) companies were studied. Use of PPE varied considerably by type: shoes (78.7%), reflective vest (44.5%), gloves (30.6%), hard hat (27.0%), nose mask (17.2%), goggles (11.3%) and hearing protection (10.8%). For all types of PPE, use was higher for workers in foreign-owned companies compared with locally-owned companies: goggles (Odds ratio [OR] 55.2), hearing protection (OR 52.0), gloves (OR 23.7), hard hat (OR 20.2), nose mask (OR 17.8), reflective vest (OR 5.3) and shoes (OR 4.1), (p<0.001 for all ORs). No site had any signage to promote PPE use.

**Conclusions:**

Majority of workers used shoes. Less than half of workers used other types of PPE and use of some types (goggles and hearing protection) was minimal. Workers in foreign-owned companies were significantly more likely to use all the seven types of PPE than locally-owned companies. Although there is still room for improvement in foreign-owned companies, locally-owned companies should be able to attain similar PPE use to that in foreign-owned companies. Necessary PPE should be provided and site supervisors should encourage workers to wear PPE when on site.

## Background

Occupational injuries cause approximately 360,000 deaths per year globally. An additional 340 million workers suffer non-fatal injuries [1]. Construction work is especially dangerous [1]. Data are not well known globally, but in high-income countries construction-related deaths account for around 15-20% of all occupational injury deaths [2, 3]. Hence, safety for construction workers is a significant issue.

Road construction has its own specific set of safety issues such as different risk exposures, different safety management strategies, and different personal protective equipment (PPE). The work takes place mostly in open environments, exposing the workers to bad weather conditions such as cold, windy, or hot environments. Many jobs are done by small and short-lived firms, working in constantly changing worksites, which makes monitoring of hazards and injuries more difficult [4–6].

Occupational safety and health is less developed in low- and middle income countries (LMICs) than in high income countries [7]. In particular, as regards construction safety in Africa, there have been only a small number of studies, primarily from East Africa and on building construction [8–12]. Gebremeskel and Yimer found a high annual prevalence of injury (33%), but also found that safety training and use of PPE lowered risk of injury [9]. Two studies looked at self-reported PPE use by construction workers in Uganda and Ethiopia, finding rates of use of any PPE of 16 – 38% [8, 12]. Izudi *et al.* found that PPE use was increased among female workers, permanent employees (as opposed to temporary or casual workers), and among people with previous knowledge of safety.

The above studies were on PPE use in building construction. In one of the few studies on safety for road construction workers in Africa, Nyende-Byakika found that use of PPE was only 14% in Uganda. Most contractors interviewed in the study thought investing in PPE was a waste of money. However, subjectively, there was a greater emphasis on safety on bigger projects, especially those that had international involvement [13].

Road construction workers should be provided appropriate protection against hazards on site. They should be provided with PPE with the approved design and construction appropriate for the exposure and the work to be performed. Section 118 Subsection (1) of the Labour Law of Ghana, ACT 651 (2003) enjoins the employer to ensure that each employee works under acceptable, harmless and healthy conditions. Subsection (2) specifies that the employer should ensure that the “work place” is free from contamination by safeguarding workers from, dust, mists, fumes, toxic gases, noxious substances, vapours and other substances or materials which could predispose them to safety or health risks. Employers are additionally responsible for the provision and maintain of adequate safety appliances, appropriate fire-fighting equipment, PPE and educate them in their use at no cost to the worker. Employers are to also prevent accidents and injury to health arising out of work by lessening the causes of hazards found in the working environment. Subsection (3) however says that it is the compulsory for each employee to use the safety appliances, fire-fighting equipment and PPE provided by the employer as per the employer’s instructions [14].

In order to address the gap in knowledge on safety for road construction workers in Ghana, we sought to determine the prevalence of several specific items of PPE among road construction workers. We also sought to determine whether posted PPE signage was adequate. Finally, we sought to determine whether type of company (locally-owned or foreign-owned) was associated with differences in PPE signage or use.

## Methods

### Setting

An institution-based cross-sectional study was conducted between 27^th^ January and 4^th^ March, 2020. The study was conducted in Ghana, a West African country of approximately 30 million people with Gross National Income of US$2,220 per capita [14]. Eighteen construction companies (13 locally-owned, 5 foreign owned) actively working on 19 different roads were purposively selected from three regions, namely, Ashanti, Ahafo and Western North. These 18 companies (14 out of 23 from Ashanti, 1 out of 2 from Ahafo and 3 out of 3 from Western North) were purposively selected because they were actively working on site. The eight companies which were not selected had either skeletal number of workers on site (3) or were on temporary break (5). The study sites selected were mostly unpaved roads with the dominant activities being construction of drains and bridges, excavation works, steel cutting and bending as well as fixing, survey activities, grading of road, hauling of laterite and boulders, road surfacing and compaction, demolition works, watering of roads, and bituminous surfacing-related activities among others (Appendix 1 Table 5).

### Profile of Study Participants

Workers at each construction companies’ sites who were working in the following crafts were selected: excavation/earth works, steel bending/erection/fixing, masons, carpenters, welders/electricians, drivers/operators, mechanics, daily labourers, site supervisors, safety officers, architects, quantity surveyors, land surveyors and civil engineers. All workers in each craft were included in the study. A total of 389 road construction workers working on site were purposively observed without their knowledge.

### Data Collection Procedure

Road construction workers of foreign- and locally-owned companies were observed without their knowledge with a checklist (Appendix 2 Table 6) to capture whether or not they wore the appropriate PPE on site. The data collectors stood at a distance or sat in cars parked near construction sites such that the observations could be done without attracting attention. The study was done from Monday to Saturday (normal working days), during the dry season (January – March). The PPE of interest were hard hat, goggles, shoes, nose masks, hearing protection, gloves and reflective vests/apparel. Shoes implied any closed toe shoes rather than sandals or other open toe shoes or bare feet. On-site posted cautionary signage was also checked. Posted safety signs on welding arc flash, scaffold safety, safety shoes, no smoking, open trench, machine safety, truck safety, keep away, open trench and fall protection, and signs advising PPE were checked. Data were collected on tablets using Open Data Kit (ODK) software. The Data collectors were given 3 days of training before the project commenced. They were directly supervised on site by the principal investigator (IKY). Data collected were checked for accuracy, completeness and uniformity by the principal investigator at the end of each day’s activity.

### Data Analysis

The dependent variables are use of various types of PPE (hard hats, goggles, shoes, nose masks, hearing protection, gloves, and reflective vests/apparel wearing). The independent variables are type of company (foreign-owned vs. locally-owned) and profession of worker. Data were cleaned and analysed using Stata 16.0 statistical software. Frequency distribution and percentage calculations were derived for the independent variables. Statistical significance was set at *p*<0.05. Relationship of dependent and independent variables was assessed using Chi-square test and Fisher’s exact test, where expected values were less than 5. Stratified analysis for the effect of company type on PPE, stratified by type of profession, was performed using Epi Info 7.1.4 statistical software.

### Ethical Considerations

The Kwame Nkrumah University of Science and Technology/Komfo Anokye Teaching Hospital Committee on Human Research, Publications and Ethics (CHRPE), approved the study. Agencies under the Ministry of Roads and Highways (MRH), namely, the Ghana Highway Authority (GHA), the Department of Urban Roads (DUR) and the Department of Feeder Roads (DFR) as well as the construction companies also gave approval for the conduct of the study at their construction sites. Information collected through the observations was anonymous and did not include name or any other identifying information on the workers.

## Results

Data were obtained from 19 sites, 14 run by locally-owned companies and 5 run by foreign-owned companies (Appendix 1 Table 5), but primarily hiring Ghanaian workers. There were no personal protective safety signage at any site studied. A total of 389 workers who were actively working on site were observed unobtrusively. Labourers formed the majority of the workers, followed by masons and drivers (Table 1). The two most common professions of workers were the same for both types of companies, with minimal differences in proportions of the other professions. There were 12 (3.1%) females and 377 (96.9%) males. The professions of the females were flagsmen (2), labourers (6), supervisors (3) and safety officers (3).

**Table 1:** Frequency of Workers by Profession and Company Type

	Foreign Owned		Ghanaian-Owned		Total	
Profession	Frequency	Percent (%)	Frequency	Percent (%)	Frequency	Percent
Labourers	88	45.4	120	61.5	208	53.5
Masons	41	21.1	27	13.9	68	17.5
Steel Benders	18	9.3	6	3.1	24	6.2
Drivers	13	6.7	22	11.3	35	9.00
Carpenters	11	5.7	9	4.6	20	5.1
Mechanics	7	3.6	3	1.5	10	2.6
Supervisors	6	3.1	7	3.6	13	3.3
Flagsmen	4	2.1	0	0	4	1.0
Safety Officers	3	1.6	0	0	3	0.8
Surveyors	3	1.6	0	0	3	0.8
Concrete Mixer Operator	0	0	1	0.5	1	0.3
**Total**	**194**	**100%**	**195**	**100%**	**389**	**100.00**

For all workers, use of PPE ranged from 78.7% for shoes to 10.8% for hearing protection (Table 2). Use of all categories of PPE was significantly higher for workers in foreign-owned companies (p<0.001 for all categories of PPE, Table 2). For workers in foreign-owned companies, use of PPE ranged from 89.7% for shoes to 21.1% for hearing protection. For workers in locally-owned companies, use of PPE ranged from 67.7% for shoes to 0.5% for each of hearing protection and goggles.

**Table 2:** Percentage Personal Protective Equipment Use by Company Type

	N	Goggles	Hard Hat	Shoes	Gloves	Hearing Protection	Reflective Vest	Nose Mask
Locally-owned	195	0.5%	4.6%	67.7%	5.1%	0.5%	25.1%	2.6%
Foreign-owned	194	22.2%	49.5%	89.7%	56.2%	21.1%	63.9%	32.0%
Total	389	11.3%	27.0%	78.7%	30.6%	10.8%	44.5%	17.2%
Odds Ratio		55.2	20.2	4.1	23.7	52.0	5.3	17.8
*p-value*		*<0.001*	*<0.001*	*<0.001*	*<0.001*	*<0.001*	*<0.001*	*<0.001*
95% CI		7.5-405.8	9.8-41.8	2.4-7.2	11.8-47.6	7.1-382.2	3.4-8.2	7.0-45.6

CI: Confidence intervals

There were also differences in use of PPE by different type of professions, with significant differences among the professions for all types of PPE (Table 3). Flagsmen and safety officers had 100% use of several types of PPE (hard hats, shoes, and reflective vests). As a whole, labourers had less than 50% use of all types of PPE, except for shoes. Use of shoes was over 90% for all professions, except for labourers and masons. Only two of the 11 professions had 90% or greater use of hard hats and only three of the professions had 90% or greater use of reflective vests. In terms of specific occupations with specific PPE needs, use of gloves by steel benders was only 20.8%. Likewise, hearing protection by mechanics and concrete mixer operators was only 40.0% and 0% respectively.

**Table 3:** Percentage Personal Protective Equipment Use by Profession

	N	Goggles (%)	Hard Hat (%)	Shoes (%)	Gloves (%)	Hearing Protection (%)	Reflective Vest (%)	Nose Mask (%)
Labourers	208	7.7	22.6	71.2	23.1	7.2	34.6	13.0
Masons	68	16.2	29.4	73.5	33.8	17.6	50.0	22.1
Drivers	35	11.0	20.0	94.3	14.0	8.6	65.0	11.0
Steel Benders	24	4.2	33.3	100	20.8	0.0	70.8	25.0
Carpenters	20	15.0	25.0	95.0	55.0	15.0	60.0	35.0
Supervisors	13	7.7%	30.8	92.3	30.8	7.7	61.5	7.7
Mechanics	10	40.0	50.0	100	60.0	40.0	90.0	40.0
Flagsmen	4	75.0	100	100	75.0	50.0	100	75.0
Safety Officers	3	66.7	100	100	66.7	66.7	100	100
Surveyors	3	0.0	66.7	66.7	0.0	0.0	33.0	0.0
Concrete Mixer Operator	1	0.0	0.0	100	0.0	0.0	0.0	0.0
*p-value*		*<0.001*	*0.002*	*<0.001*	*0.01*	*<0.001*	*<0.001*	*0.005*
**Total**	**389**							

It is possible that the difference we observed in PPE use between company types might be explained by the higher percentage of labourers working in locally-owned companies. Hence, we further evaluated PPE use by company type, looking at the differences stratified by labourers vs. all other professions (Table 4). The differences between companies persisted, with statistically higher use of all types of PPE by workers in foreign-owned companies than in locally-owned companies, both for labourers and for all other professions. Odds ratios adjusted for type of profession were significantly higher than one for all types of PPE and were minimally different from the univariate odds ratios (Table 2). Adjusted ORs for effect of company type: goggles (57.5), hard hat (20.4), shoes (3.8), gloves (24.3), hearing protection (43.8), reflective vest (4.8), and nose mask (17.8).

**Table 4:** Use of Personal Protective Equipment by Type of Company and Category of Worker

	N	Goggles	Hard Hat	Shoes	Gloves	Hearing Protection	Reflective Vest	Nose Mask
Labourer								
Locally-owned	120	0.8%	5.8%	59.2%	5.0%	0%	15.0%	3.3%
Foreign-owned	88	17.0%	45.5%	87.5%	61.4%	15.9%	61.4%	26.1%
*p value*		*<0.001*	*<0.001*	*<0.001*	*<0.001*	*<0.001*	*<0.001*	*<0.001*
Total	208							
All other professions								
Foreign-owned	106	26.4%	52.8%	91.5%	51.9%	25.5%	66.0%	36.8%
Locally-owned	75	0%	2.7%	81.3%	5.3%	1.3%	41.3%	1.3%
*p value*		*<0.001*	*<0.001*	*0.043*	*<0.001*	*<0.001*	*<0.001*	*<0.001*
Total	181							
Adjusted Odds Ratio*		57.5	20.4	3.8	24.3	43.8	4.8	17.8
95% CI		7.3-451.8	9.8-42.8	2.2-6.6	11.9-49.4	6.2-311.3	3.1-7.4	6.8-46.2
*p value*		*<0.001*	*<0.001*	*0.043*	*<0.001*	*<0.001*	*<0.001*	*<0.001*

*Mantel-Haenszel adjusted odds ratio for the effect of company type (foreign-owned vs locally-owned) on use of personal protective equipment, adjusted for type of profession (labourer vs. all other professions).

CI: Confidence intervals

## Discussion

This study sought to determine the prevalence of use of seven types of PPE by road construction workers in Ghana. It also sought to determine the presence or absence of signage related to use of PPE. No site had any type of signage cautioning workers to use PPE of any kind. PPE use was moderate and ranged from most workers using shoes to very few workers using goggles or hearing protection. PPE use varied among the professions, being higher for flagsmen and safety officers and lower for labourers. PPE use varied dramatically by type of company. Workers in foreign-owned companies used all of the seven types of PPE significantly more often than workers in locally-owned companies.

In one of the few prior studies on the topic of PPE use by road construction workers in Africa, Nyende-Byakika studied four road construction sites in Uganda [13]. Interviews with workers and managers were conducted. Self-reported use of PPE ranged from 0% to 20% for the different sites, averaging 14%. Subjectively, many contractors indicated that they felt investing in PPE was not warranted. There have been several other studies that reported PPE use and other aspects of safety among building construction workers in Africa, including Ethiopia and Uganda. Self-reported PPE use ranged from 16% to 38% [8–12].

Prior studies from Ghana have also looked at safety in building construction, but not yet road construction. Ofosu *et al.,* found that PPE use among building construction workers was not encouraging (15% for helmets, 20% goggles among welders and 15% for safety shoes) [17]. Agyekum *et al.,* evaluated factors that influenced the performance of safety programmes in 60 Ghanaian building construction firms, identifying 13 elements that improved safety programmes, such as providing safety managers on site and providing safety orientation training [18].

This study thus adds to the literature on PPE use by constructions workers in Ghana, and especially by road construction workers. It uses direct observation of PPE use, in comparison to the self-report used by the above studies and it reports on use of specific types of PPE. The types of PPE evaluated in the current study protect against injuries (e.g. hard hats and safety boots), as well as lung damage from dust and silica inhalation (e.g. nose masks), and hearing loss (e.g. hearing protection). The effectiveness of the various types of PPE is well documented [15, 16]. It is also notable that one of the above studies from Ethiopia reported an annual prevalence of injury of 32.6%. This was decreased by half by both PPE use and safety training [9].

It is also notable that the current study showed a large difference between foreign-owned and locally-owned companies. Foreign-owned companies might be slightly better resourced than locally-owned companies. The improved PPE use in foreign-owned companies may also be the effect of different management practices. There have been several reports of the effect of different management practices. For example, in Addis Ababa, Ethiopia, Alemu *et al.* reported that prior training in PPE use and presence of on-site supervisors both increased use of PPE around five-fold each [12]. Nyende-Byakika looking specifically at road construction workers in Uganda reported that, subjectively, workers felt that there was a bigger emphasis on safety by the same companies on bigger projects that had international involvement [13]. A study conducted in Kenya by Mitullah *et al.* revealed differences in safety practices depending on the type of worker. About 70% of casual workers were not provided with welfare-related facilities and safety materials at most project sites [17].

The current study showed that none of the 19 sites visited had signage about PPE use. One other study on PPE use in road construction in Africa in the literature reported that all four sites studied had signage to warn passing vehicles to slow down, but did not comment on signage about PPE [13].

It is also important to note that similar issues on construction safety pertain in many other low- and middle-income countries. For example, a study of 23 building construction companies in Jordan revealed that only nine had safety policies and most construction sites visited had no evidence of health and safety practices in place, such as posted safety signs or PPE in use [19]. In Honduras, interviews with 108 building construction workers and 18 managers revealed that management did not feel it was in their interests to improve safety, only 25% of companies had safety programmes, and workers rarely used PPE [20]. In Indonesia, a study of 200 construction workers at an airport renovation project showed that only 25% used PPE consistently [21].

The study had the following limitations: First, construction sites were purposively selected, rather than randomly. This was necessary as permission from the government highway authorities and the construction companies was needed in advance. Second, as observations were carried out anonymously and unobtrusively with no interactions with the workers, the only information on the worker’s characteristics that could be gathered was gender and profession. This limited the ability to analyse associations among other worker characteristics and PPE use. Third, observations of PPE use were one time and it was not possible to ascertain usage over time during the course of a shift. Fourth, observations were done only in the middle belt of the country and during the dry season, limiting the ability to make nationwide conclusions or to assess differences in PPE in different seasons, respectively. Finally, it was not possible to ascertain the quality of the PPE observed. This was especially an issue for shoes. It would have been ideal to assess whether workers were wearing steel-toed safety shoes, but with the unobtrusive observations, the best that could be done was to distinguish full shoes from sandals and bare feet. Despite these limitations, the study has the strengths of being one of the first studies to address PPE use in road construction workers in Africa, of having a large sample size, and of using direct observation, which is more reliable than self-report.

## Conclusion

This study found that PPE use among road construction workers in Ghana was incomplete and varied with type of PPE and profession of worker. Use ranged from most workers using shoes to very few workers using goggles or hearing protection. PPE use was highest among for flagsmen and safety officers and lowest for labourers. A notable finding of the study was the large differences in PPE use between company types. Workers in foreign-owned companies used all of the seven types of PPE significantly more often than workers in locally-owned companies. Although there is still room for improvement in PPE use by foreign-owned companies, a feasible next step for occupational safety for road construction in Ghana is to increase use of PPE among locally-owned companies to that in foreign-owned companies. This could be achieved by low-cost and simple means such as companies hiring safety managers who have professional training and by providing needed PPE to workers. Site supervisors should also encourage workers to wear PPE when on site.

## Data Availability

The datasets used and/or analysed during the current study are available from the corresponding author on reasonable request.

## Appendix 1

Table 5, Table 6

**Table 5:** Study sites

Road Visited	Road Type	Purpose
**Foreign-Owned Construction Company Sites**		
Ahensan to Chirapatre	Urban road	Rehabilitation and upgrading
Terchire to Adrobaa	Feeder road	Gravel to bitumen
Mpasatia to Bedaabour	Feeder road	Gravel to bitumen
Kentinkrono township	Urban road	Gravel to bitumen
Onwe-Achinakrom-Deduako-Kwaso to Donyina	Urban road	Gravel to bitumen
**Locally-Owned Construction Company Sites**		
Kumasi Airport roundabout to Buokrom	Urban road	Rehabilitation
Fumesua township	Urban road	Gravel to bitumen
Sefwi Wiawso township	Urban road	Rehabilitation
Chirano to Akoti Junction	Feeder road	Gravel to bitumen
Wiawso College of Education area roads	Urban road	Gravel to bitumen
Daban to Ampeyoo	Urban road	Rehabilitation
Pemenase to Ankaase Lakeside	Feeder road	Gravel to bitumen
Kokofu through Amakom to Lake Bosomtwe	Feeder road	Gravel to bitumen
Ahenkro to Tetrem	Feeder road	Gravel to bitumen
Bekwai township	Urban road	Upgrading
Krapa township	Urban road	Gravel to bitumen
Achinakrom area	Feeder road	Gravel to bitumen
Main Nyinahin road to Adobewura	Feeder road	Gravel to bitumen
KNUST Police Station area roads	Urban road	Rehabilitation

**Table:** Table 6

No.	Posted PPE Safety Signage		Yes	No	Remark (s)
1	Availability of PPE Safety signage	Ear protection			
		Eye protection			
		Welding			
		Scaffold/Ladder			
		Crane safety			
		Fall protection			
		Gas cylinder			
		Gloves required			
		Safety shoes			
		No smoking			
		Open trench			
		Head protection			
		Electrical/high voltage			
		Flammable materials			
		Forklift			
		Keep away			
		Machine safety			
		Personal Protection			
		Truck safety			
		Watch your step			
		Chemical hazard			
		Confined space			
		Safety awareness			
		Safety tape			
		Ladder scaffold safety			
		Flash lights (For Night Workers)			
	**Use of PPE?**		**Yes**	**No**	
2	Hard hats or helmets				
3	Goggles/face shield (for Welders)				
4	Boots/shoes				
5	Ear plug/muff				
6	Nose mask				
7	Hand gloves				
8	Reflective vest/apparel				

## Appendix 2

On-Site Personal Protective Equipment Signage and Use by Road Construction Workers in Ghana: A Comparative Study of Foreign- and Locally-Owned Companies

(Unobtrusive Observational Study at Road Construction Sites)

Name of Institution: ……………………………………………………………………………………………………………

Company Type: Foreign-Owned (………) Locally-Owned (……….)

Profession/Artisan:

## Abbreviations

CHRPE: Committee on Human Research, Publications and Ethics
DFR: Department of Feeder Roads
DUR: Department of Urban Roads
GHA: Ghana Highway Authority
LMIC: Low- and Middle Income Countries
ODK: Open Data Kit
PPE: Personal Protective Equipment
